# Health-related quality of life assessed by EQ-5D-5L and its determinants among Chinese adults

**DOI:** 10.3389/fpubh.2024.1383781

**Published:** 2024-09-11

**Authors:** Gengliang Bai, Jiawen Zhang, Yijun Chen, Lejing Cao, Yong Yang, Chao Jiang

**Affiliations:** ^1^School of Health Economics and Management, Nanjing University of Chinese Medicine, Nanjing, China; ^2^School of International Pharmaceutical Business, China Pharmaceutical University, Nanjing, China; ^3^Affiliated Hospital of Nanjing University of Chinese Medicine, Nanjing, China

**Keywords:** quality of life, EQ-5D-5L, EQ-VAS, health status, China

## Abstract

**Background:**

Due to the rising standard of living and advances in public health and medical care in recent years in China, the health-related quality of life (HRQoL) has been increasingly acknowledged as an important part of health management of adults. This study aimed to analyze the HRQoL of Chinese adults and identify the influencing factors, proposing specific recommendations for improvement.

**Methods:**

A cross-sectional study was conducted among 1,291 selected adults from four provinces spanning different regions in China aged ≥18 years from July 2021 and January 2022. The EuroQol-5D-5L (EQ-5D-5L) was used to conduct the HRQoL survey, and a general questionnaire was administered to collect demographic characteristics, general information, and health behaviors of participants. The health utility value was measured, and one-way analysis of variance was performed. The Tobit regression model was employed to analyze the factors influencing the HRQoL of Chinese adults.

**Results:**

The mean health utility values and visual analog scale scores for adults were 0.9400 ± 0.1197 and 84.09 ± 14.392, respectively. Notably, 60.3% of respondents reported no difficulties in any of the five dimensions of EQ-5D. However, a substantial proportion faced challenges in anxiety/depression (27%) and pain/discomfort (26.2%). Tobit regression model revealed that age, marital status, educational level, diet, sleep, mental state, mood, and chronic diseases significantly impact the HRQoL of Chinese adults.

**Conclusion:**

The HRQoL among Chinese adults is generally satisfactory, but pay particular attention on areas such as pain, psychological anxiety, chronic diseases, and negative emotions is needed. The factors such as stress associated with marriage and the demands of high-skilled occupations might influence the overall health of the population. According to our findings, public health strategies to improve HRQoL should be developed to promote relatively healthy environments and lifestyles for older adults. Moreover, proactive measures are crucial for mitigating the potential health impacts associated with marital stress and high-skilled employment.

## 1 Introduction

During the past 30–40 years, the standard of living, health care, and public health have significantly improved as part of the economic reforms in China. Healthy China 2030 was developed in 2016 with the aims of increasing the life expectancy and improving the HRQoL in all Chinese people. With the implementation of Healthy China 2030, there is a notable shift from a “disease-centered” to a “health-centered” approach, with individuals increasingly focusing on various aspects of their overall health. However, Chinese adults are also confronted with new challenges related to promoting the physical and mental wellbeing and reducing health inequalities (1). Chronic diseases, unhealthy lifestyles, and various other factors have been identified as significant contributors to the deteriorating health status and reduced life expectancy among the Chinese population (2). A comprehensive survey revealed that a substantial proportion of Chinese adults, approximately 83%, experience varying degrees of mental stress (3). Quality of life (QOL) has become established as a significant concept and target for research and practice in the fields of health and medicine. Health-related QOL (HRQoL) was developed by combining the theory of QOL and the practice of medicine. HRQoL is often described as: “A term referring to the health aspects of QOL, generally considered to reflect the impact of disease and treatment on disability and daily functioning; it has also been considered to reflect the impact of perceived health on an individual’s ability to live a fulfilling life” (4). HRQoL is a cornerstone in the evaluation of modern medicine, healthcare practice, and other medical interventions (5). As an effective tool for economic evaluation, research, and health interventions, HRQoL assesses the impact of health conditions on the daily lives of individuals and is useful in describing the life experiences of people as it relates to their health (6). Furthermore, HRQoL serves not only as a reflection of an individual’s comprehensive health status but also as an essential measure of the collective well-being of adults within a region. The indicators of poor HRQoL are strong predictors of mortality, independent of other behavioral, medical, and psychosocial risk factors (7). National HRQoL monitoring can assess population changes over time and identify unmet population health needs. Therefore, measuring the HRQoL in different populations is essential for informing the assessment of health outcomes and the development of healthcare services.

In recent years, HRQoL has received worldwide attention, and several multidimensional health status classifications have been increasingly used to describe and evaluate HRQoL in China. In recent years, research on the HRQoL of Chinese adults has focused on a specific region in China such as Northern China (1), Western China (8), Hainan (9), Shenyang (10), Hong Kong (11), Henan (12), Shaanxi (13), Southern Jiangsu (14), Zhejiang, and Qinghai (15). In addition, based on China’s specific national conditions, many local studies on the HRQoL of the population have focused on groups such as the older adults, rural adults, people with chronic diseases (9, 16), and adolescents (17). Some studies have demonstrated that the HRQoL among the older adults in China is superior to that in Western countries (18). In Western countries, research has focused mainly on measuring the HRQoL of serious diseases, such as cancer (19), often involving large national samples (6, 20). Some studies have also examined the HRQoL of farmers, revealing that the HRQoL of individuals is not optimal. More than half of the participants reported experiencing moderate or extreme pain/discomfort, and almost a third reported some or severe problems with anxiety/depression (21).

Some earlier studies have evaluated the influencing factors of HRQoL. Dong et al. (22) utilized data from the 2010 China Chronic Disease and Risk Factor Surveillance to assess the HRQoL of Chinese adults. They found that college graduates and those with a higher educational attainment had the highest mean number of mentally unhealthy days (22). Furthermore, with the rising living standards and advancements in public health and medical care, the life expectancy of Chinese adults is increasing annually and the overall HRQoL is improving. However, this is also accompanied by an increasing prevalence of chronic diseases, such as hypertension, diabetes, and obesity, which is inversely associated with the HRQoL (23). Previous studies have demonstrated that gender, age, education, marital status, region of residence, urban and rural factors, diet, sleep quality and chronic diseases influence the HRQoL of Chinese adults (10, 24).

Early studies focusing on the HRQoL of Chinese adults primarily relied on simple questionnaire-based surveys. As a result, studies on the overall health status of Chinses adults are lacking. The determinants of HRQoL have varied across studies and have not been reassessed since 2016. EQ-5D is an HRQoL questionnaire developed by the EuroQol Group, which is widely used for measuring the HRQoL of patients with various diseases (25). The Chinese version of ED-5D-5L was developed and validated for use in mainland Chinese residents (26). Developed by Luo et al. (27), its score is estimated on the basis of health preferences of urban adults of China, shows good reliability and validity (28, 29). Therefore, this study aimed to assess the HRQoL of adults living in Eastern, Central, and Western parts of China using EQ-5D-5L, as well as identify its influencing factors. This is the first study to utilize the EQ-5D-5L to evaluate the HRQoL and its determinants among adults from different regions of China. Our results can provide useful information for policymakers.

## 2 Materials and methods

### 2.1 Study design and participants

This cross-sectional study was conducted among a demographically representative sample of Chinese adults aged ≥18 years from July 2021 and January 2022. Using the formula *n* = (u_α_σ/δ)^2^ (where α is the type I error and u_α_ is the standard normal distribution), with α = 0.05, u_α_ = 1.96, two-sided significance of 0.05, HRQoL scoring error < 2.5 points (δ), standard deviation σ = 30, and response rate of 80% (1), a sample size of 700 was determined sufficient for this survey. The data for this study were gathered from HRQoL field surveys conducted in selected provinces across China, namely Jiangsu Province in the East, Henan Province in the Central region, and Guangxi and Guizhou Province in the West. This geographical spread aimed to capture diverse economic development levels within China. Respondents were recruited from at least three different cities within each province using a stratified convenience sampling method. Taking Jiangsu Province as an example, the participants were selected as follows: first, we selected Nantong City from Jiangsu Province. Second, the Qidong district was selected from Nantong City. Third, Huilong town was selected as the study area. Finally, participants were selected from publicly accessible places such as parks, shops, streets, hospitals, and university campuses, as well as private locations such as participants’ residences.

Participants included in the study met the following criteria: (1) aged ≥18 years; (2) Chinese nationality and residing in mainland China; (3) literate and free from cognitive impairments such as dementia; and (4) willing to voluntarily participate in the survey. Trained interviewers guided participants through the survey process, addressing any queries that arose. Questionnaires with missing responses or incomplete sections due to discontinuation were excluded from the analysis. A total of 1,353 questionnaires were collected, of which 1,291 were deemed valid. The study included 717 participants from Jiangsu Province, 301 from Henan Province, 192 from Guangxi Province, and 81 from Guizhou Province.

### 2.2 Data collection

Face-to-face interviews were conducted, which utilized a self-designed questionnaire comprising three sections: (1) Demographic characteristics, including gender, age, marital status, education level, and prevalence of chronic diseases; (2) General information and health behaviors, covering topics such as sleep condition, mental state, mood, and others; and (3) EQ-5D scale. The questionnaire was intended for self-completion, and paper copies were distributed to respondents. Data were collected through one-on-one, face-to-face personal interviews conducted by a team comprising interviewers and investigators from Nanjing University of Chinese Medicine. Each team, consisting of local students serving as interviewers, was supervised by principal investigators who conducted daily quality checks on the collected data. The study protocol was approved by the Institutional Review Board of Affiliated Hospital of Nanjing University of Chinese Medicine.

Prior to data collection, interviewers underwent standardized training to ensure a consistent understanding of each questionnaire item. Simulated interviews were conducted to familiarize interviewers with potential issues and provide explanations as needed. Following training, interviewers developed a unified interpretation of each questionnaire item to ensure consistent survey administration and accurate results. This approach aimed to prevent misunderstandings resulting from inappropriate survey methods.

Interviewers from different provinces selected adults in designated cities as survey participants. The interview process followed a structured format: First, interviewers explained the survey’s purpose and content to respondents and obtained their consent. Second, the respondents were asked to fill out the questionnaire themselves. For those experiencing difficulty, interviewers provided one-on-one assistance in selecting options that closely matched their preferences. Third, interviewers checked each completed questionnaire for missing items or obvious issues upon collection and reminded respondents to supplement or adjust their responses promptly.

### 2.3 HRQoL measurement

Previous research has demonstrated that the EQ-5D-5L offers superior collective and group validity compared to the 3 L version. It exhibits higher sensitivity and precision in measuring health conditions and significantly reduces the “ceiling effect” (30, 31). This study employed the EQ-5D-5L as the assessment tool for HRQoL to calculate the health utility. License to use the EQ-5D-5L was obtained from the EuroQol Research Foundation. The health utility, ranked on a 1–0 full health-death scale, can be calculated by a value set for a range of possible health states described by the health state classification system (32). The EQ-5D-5L is currently the officially recommended version and features a utility scoring formula applicable for calculating adults’ health utility values in mainland China (27).

The EQ-5D-5L consists of a five-dimensional descriptive system questionnaire (mobility, self-care, usual activities, pain/discomfort, and anxiety/depression) and a Visual Analogue Scale (EQ-VAS) (33). Therefore, this scale comprises 3,125 potential health states, with 11,111 being the best health state (full health) and 55,555 being the worst (34). Each health state is characterized by the EQ-5D-5L classifier, denoted by a unique five-digit code, with each digit corresponding to one of the five dimensions. For example, “12,111” represents a state where a person experiences slight problems with self-care but is free from challenges in the other four dimensions. Conversely, “54,123” indicates a state where a person is unable to walk, faces severe difficulties in washing or dressing, encounters no issues with routine activities, experiences slight pain or discomfort, and deals with moderate levels of anxiety or depression. This study depicts profiles of EQ-5D-5L domains according to frequencies of each item response and the cumulative frequency of health states, as well as discussed the distribution of VAS.

### 2.4 Statistical analysis

IBM SPSS Statistics (version 25.0) and Stata (version 14.1) were utilized for statistical analysis. Descriptive statistical analysis was employed to characterize the basic profile of the participants and the EQ-5D results. Additionally, one-way analysis of variance (ANOVA) was conducted to assess factors potentially influencing the HRQoL. As over half of the respondents had a health utility value of 1, resulting in data truncation, Tobit regression was employed to investigate the factors influencing the health utility value of the adults (9), with statistical significance set at *p* < 0.05. In this study, the EQ-5D health utility value was considered the dependent variable, while demographic characteristics (such as residential area, gender, and household registration), personal health behaviors (including diet, sleep, and mental and emotional states), and health conditions (presence of chronic diseases) were included as independent variables in the regression model.

## 3 Results

### 3.1 Demographic and health-related characteristics

Table 1 presents the general characteristics of the respondents. The total sample comprised 1,291 respondents, with ages ranging from 18 to 92 years and a mean age of 41.10 ± 16.03 years. Among them, 69.6% were married, and the majority had educational backgrounds concentrated in middle school/technical school and college/undergraduate studies. There were 347 participants with chronic illnesses, accounting for 26.7% of the total sample. Among these patients, 64 respondents had two or more chronic diseases, representing 28.4% of the overall number of individuals with chronic conditions.

**Table 1:** EQ-5D score among Chinese residents with different demographic characteristics.

Variables	*n* (%)	EQ-5D score		
		*x̄*	*t/F*	*p*
Area of residence			6.284	0.002
East	717 (55.5%)	0.9299		
Center	301 (23.3%)	0.9479		
West	273 (21.1%)	0.9578		
Gender			0.451	0.652
Male	621 (48.1%)	0.9415		
Female	670 (51.9%)	0.9385		
Household registration			−0.279	0.781
Countryside	634 (49.1%)	0.9390		
Cities and towns	657 (50.9%)	0.9409		
Age			14.290	<0.001
18–34	499 (38.7%)	0.9593		
35–54	528 (40.9%)	0.9356		
≥ 55	264 (20.4%)	0.9122		
Marital status			47.375	<0.001
Unmarried	342 (26.5%)	0.9651		
Married	898 (69.6%)	0.9385		
Divorced and widowed	51 (4.0%)	0.7966		
Educational			8.280	<0.001
Primary or lower	153 (11.9%)	0.9006		
Junior or senior high school	505 (39.1%)	0.9399		
College	574 (44.5%)	0.9526		
Postgraduate and above	59 (4.6%)	0.9206		
Diet			52.268	<0.001
Normal	982 (76.1%)	0.9574		
Reduced appetite and food intake	300 (23.2%)	0.8882		
Inability to eat properly	9 (0.7%)	0.7626		
Sleep condition			74.061	<0.001
Normal	940 (72.8%)	0.9622		
Cannot fall asleep easily or wake up easily after sleeping	315 (24.4%)	0.8883		
Inability to sleep properly	36 (2.8%)	0.8111		
Psychological condition			88.201	<0.001
Full of energy, not feeling tired	691 (53.5%)	0.9667		
Average spirit, sometimes tired	565 (43.8%)	0.9201		
Listless and tired all the time	35 (2.7%)	0.7324		
State of mind			133.646	<0.001
Normal	1,130 (87.5%)	0.9571		
Depressed, reduced speech	143 (11.1%)	0.8406		
Pessimism, all is lost	18 (1.4%)	0.6578		
Number of chronic conditions			55.996	<0.001
0	945 (73.2%)	0.9595		
1	282 (21.8%)	0.8961		
≥2	64 (5.0%)	0.8451		

Based on the Chinese value set, the health utility value of the adults was 0.9400 ± 0.1197, with 778 individuals having a health utility value of 1, comprising 60.3% of the interviewees. Notably, over half of the adults reported no problems across the five dimensions of the EQ-5D, indicating the presence of a ceiling effect in the practical application of this scale. This effect was further substantiated through statistical analysis.

Significant disparities in health utility values were observed among adults residing in different areas, belonging to various age groups, exhibiting diverse marital statuses, possessing varying educational levels, dietary habits, sleep patterns, mental states, moods, and those suffering from distinct types of chronic diseases (*p* < 0.05). However, the differences between urban and rural areas were found to be insignificant (*p* > 0.05). Geographically, health utility values for adults in the east, mid-west, and west exhibited a gradual increase, with the east being slightly below the overall average. Concerning age, health utility values declined progressively with increasing age. Marital status also played a role, with divorced or widowed adults displaying a lower health utility value < 0.8.

Educational levels showed an interesting trend, with health utility values gradually increasing for adults with primary school or below, secondary school/technical school, and college/bachelor’s degree. However, there was an inflection point at the college/bachelor’s degree level, beyond which values started to decrease for adults with a master’s degree or above. Various living habits were found to influence health utility values, with a more regular appetite and food intake, normal sleep patterns, a fuller mental state, and stable emotions correlating with higher health utility values. Regarding the prevalence of chronic diseases, non-diseased adults demonstrated significantly higher health utility values compared to their diseased counterparts. These findings underscore the multifaceted impact of demographic and lifestyle factors on adults’ HRQoL.

### 3.2 Overview of the situation

**Figure 1:**
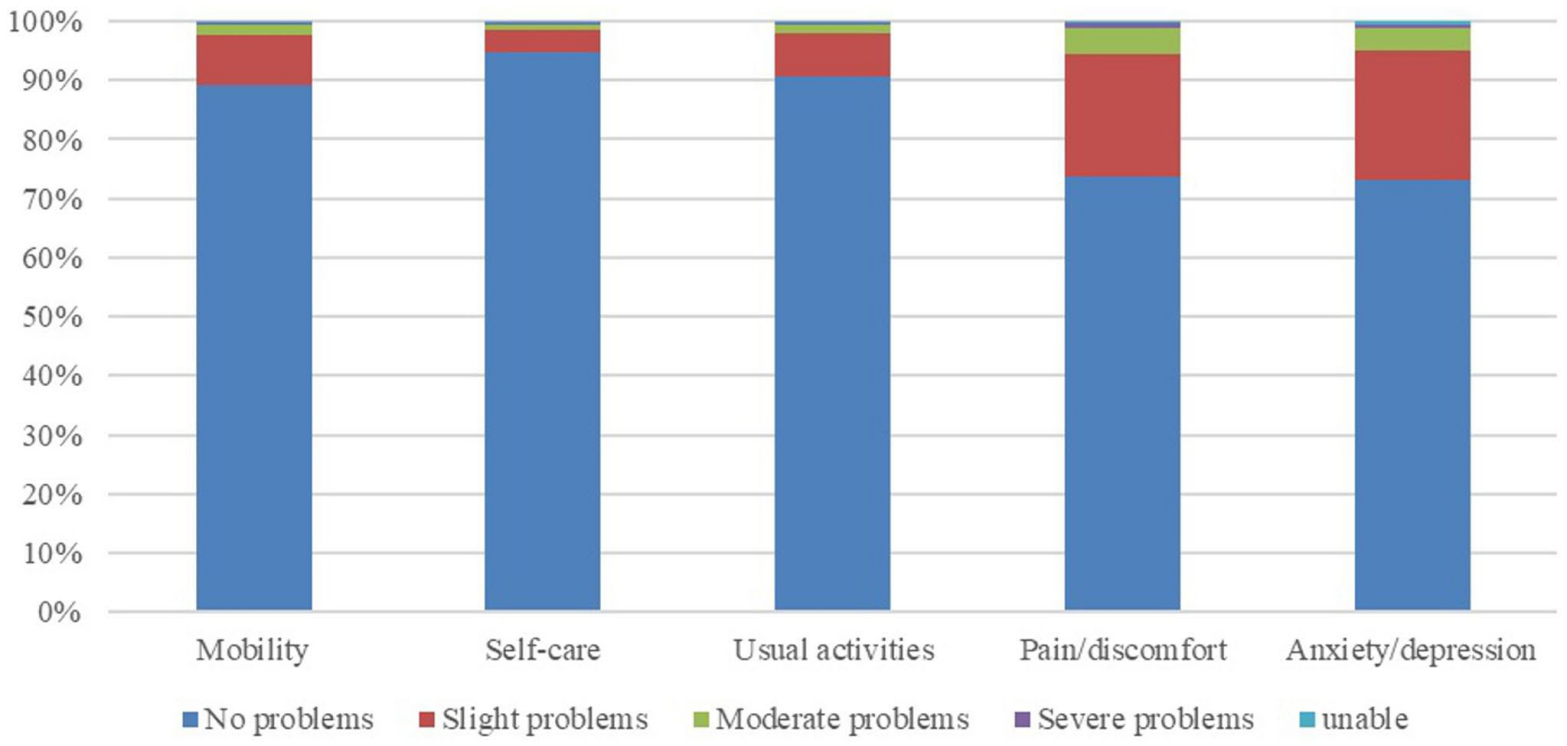
HRQoL percentage plot based on EQ-5D-5L.

In the EQ-5D’s five dimensions, “anxiety/depression” and “pain/discomfort” emerge as the dimensions with the highest prevalence of difficulties. Approximately 27 and 26.2% of respondents reported experiencing at least slight difficulty in these dimensions. Following closely is the dimension of “mobility,” with 10.9% of respondents encountering difficulties. Notably, “self-care” and “usual activities” exhibited lower levels of difficulty, with fewer than 10% of respondents facing challenges, as illustrated in Figure 1.

### 3.3 Cumulative frequency of EQ-5D health states

Among the 1,291 respondents in this study, 778 individuals reported no difficulties in any of the five dimensions, indicating a health status of “11,111,” constituting 60.3% of the total sample. In contrast, 39.7% of the adults encountered varying degrees of problems in at least one dimension, with pain and anxiety being the primary issues. Specifically, 925 respondents experienced difficulties in only one dimension, accounting for 71.6% of the total. Notably, only 17 individuals faced severe difficulties in one dimension, represented by a health status indicating at least one “5.” This overall pattern suggests a generally high health status among the interviewed adults, as detailed in Table 2.

**Table 2:** Cumulative frequency of EQ-5D-5L health states.

State of health	Frequency (%)	Cumulative frequency (%)
11,111	778 (60.3)	778 (60.3)
11,112	124 (9.6)	902 (69.9)
11,121	89 (6.9)	991 (76.8)
11,122	78 (6.0)	1,069 (82.8)
11,113	12 (0.9)	1,081 (83.7)
21,122	12 (0.9)	1,093 (84.6)
21,121	11 (0.9)	1,104 (85.5)
21,111	9 (0.7)	1,113 (86.2)
21,222	9 (0.7)	1,122 (86.9)
55,555	0 (0.0)	1,291 (100)

### 3.4 EQ-VAS

**Figure 2:**
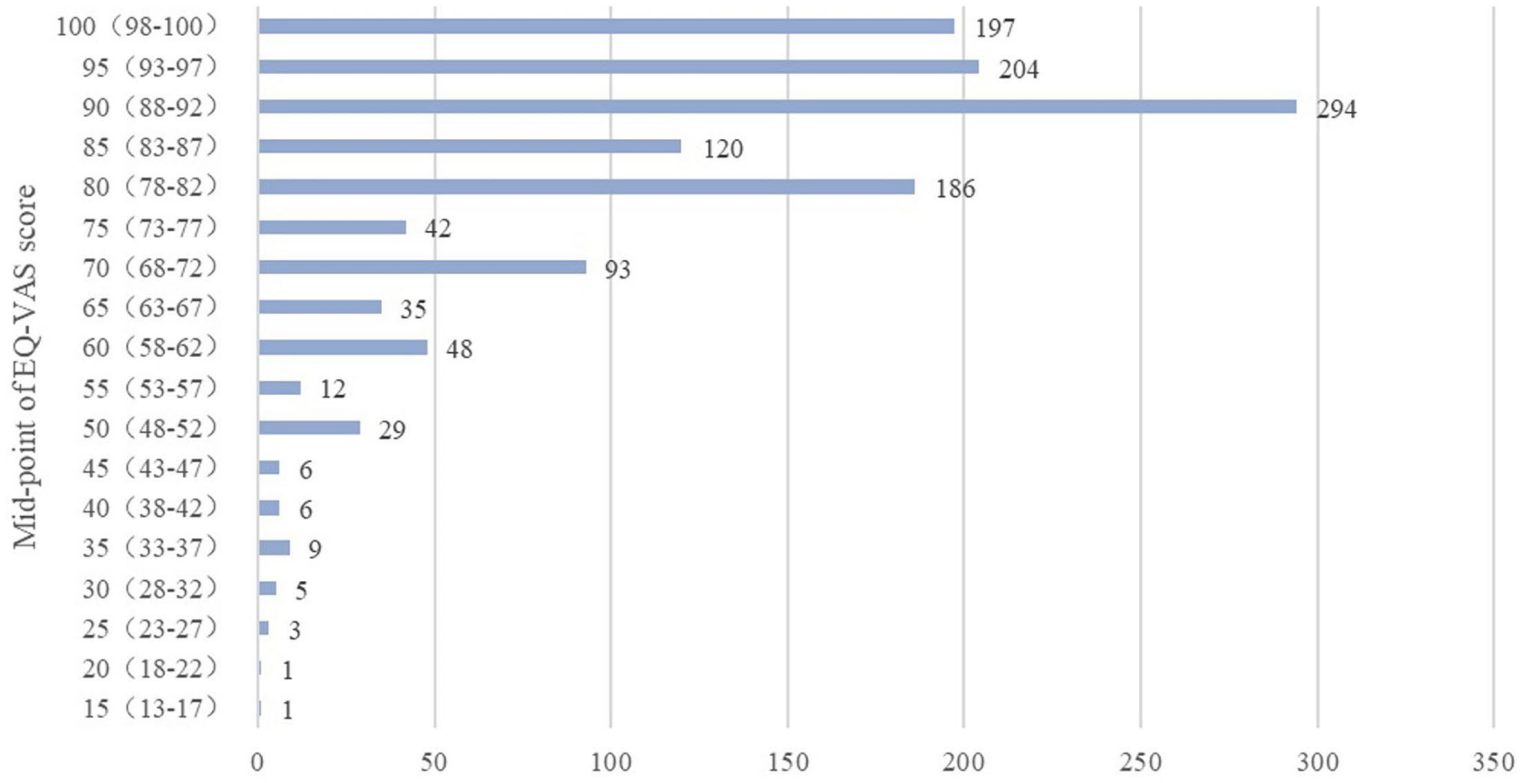
Distribution of VAS scores.

The VAS score serves as a self-assessment, reflecting the respondent’s perceived health status on the day of the survey, ranging from 0 to 100. A score of 0 represents the worst imaginable health status, while 100 signifies the best. The collective VAS score for the 1,291 respondents in this study was 84.09 ± 14.392. Among them, 147 individuals achieved a perfect score of 100, constituting 11.3% of the overall respondents. Additionally, 74 respondents scored below 60, with the lowest score recorded as 15. Given the prevalence of VAS scores being multiples of 5 and 10, this paper redefines the data ranges to align with midpoints (multiples of 5 or 10) (35). As depicted in the figure, the concentration area of VAS self-scores is within the 80–90 segments, and the distribution of scores is visually presented in Figure 2.

### 3.5 Associated factors of HRQoL

In this study, the health utility value served as the dependent variable, while variables exhibiting significant differences in the one-way ANOVA presented in Table 1 were employed as independent variables for inclusion in the Tobit regression model. This approach aimed to scrutinize the factors influencing the health utility value of the population. The results indicate that the *p*-value of the likelihood ratio test is 0.000 (<0.05), suggesting that incorporating nine explanatory variables enhances the model’s efficacy. This signifies the validity of the model construction.

Among these variables, there exists a statistically significant difference (*p* < 0.05) in the health utility value of the population based on marital status, educational level, dietary habits, sleep patterns, mental state, mood, and the prevalence of chronic diseases (Table 3). In this study, no differences in HRQoL were observed among adults from different regions. Specifically, unmarried status, normal appetite and food intake, and normal sleep conditions were identified as protective factors for HRQoL. Conversely, having a postgraduate degree or above, experiencing listlessness and tiredness consistently, harboring pessimistic feelings such as “all is lost,” and having chronic diseases were identified as risk factors for HRQoL.

**Table 3:** Tobit regression of health utility value of Chinese residents.

Term	*B*	SE	*t*	*p*	95% CI
Constant (math.)	0.398	0.086	4.61	0.000	(0.029, 0.037)
**Area of residence (Reference: East)**					
Center	0.002	0.016	0.15	0.882	(−0.029, 0.033)
West	0.020	0.017	1.13	0.257	(−0.014, 0.054)
**Age (Reference: 18–34)**					
35–54	−0.015	0.019	−0.79	0.429	(−0.053, 0.022)
≥55	0.003	0.023	0.15	0.884	(−0.042, 0.049)
**Marital status (Reference: Unmarried)**					
Married	−0.012	0.020	−0.61	0.539	(−0.052, 0.027)
Divorced and widowed	−0.125	0.034	−3.69	<0.001	(−0.191, −0.058)
**Educational level (Reference: Primary or lower)**					
Junior or senior high school	0.046	0.020	2.27	0.023	(0.006, 0.086)
College	0.042	0.023	1.85	0.065	(−0.003, 0.086)
Postgraduate and above	−0.034	0.035	−0.99	0.324	(−0.102, 0.034)
**Diet (Reference: Inability to eat properly)**					
Reduced appetite and food intake	0.089	0.063	1.41	0.160	(−0.035, 0.212)
Normal	0.157	0.063	2.49	0.013	(0.033, 0.281)
**Sleep condition (Reference: Inability to sleep properly)**					
Cannot fall asleep easily or wake up easily after sleeping	0.039	0.034	1.14	0.256	(−0.028, 0.107)
Normal	0.111	0.035	3.19	0.001	(0.043, 0.178)
**Psychological condition (Reference: Listless and tired all the time)**					
Average spirit, sometimes tired	0.139	0.033	4.16	<0.001	(0.073, 0.204)
Full of energy, not feeling tired	0.197	0.035	5.69	<0.001	(0.129, 0.265)
**State of mind (Reference: Pessimism, all is lost)**					
Depressed, reduced speech	0.172	0.047	3.66	<0.001	(0.080, 0.265)
Normal	0.277	0.045	6.17	<0.001	(0.189, 0.365)
**Number of chronic conditions (Reference: 0)**					
1	−0.063	0.016	−3.88	<0.001	(−0.094, −0.031)
≥2	−0.119	0.028	−4.23	<0.001	(−0.175, −0.064)

## 4 Discussion

This study is the first to utilize the EQ-5D-5L to measure the HRQoL and determinants of adults in the Eastern, Central, and Western regions of China. The study results revealed that our adults had a mean health utility value of 0.9400, which is similar to the mean utility index of urban China (0.945) (1), Japan (0.955) (36), and Singapore (0.95) (37), but was higher than that of United States (0.815) (38), Slovenia (0.808) (39), New Zealand (0.847) (40), Moscow (0.907) (41), Germany (0.84) (42), Hong Kong (0.919) (11), Australia (0.86) (20), Vietnam (0.91) (43), and Poland (0.888) (44). Notably, 60.3% of the adults reported no difficulties in any of the five dimensions of the EQ-5D, a proportion similar to Vietnam (67.4%) (43), Japan (55%) (36), and urban China (54%) (33), but were significantly higher than that of New Zealand (40), Germany (45), Poland (44), Canada (46), and Australia (20), ranging from 20.8 to 46.0%. However, it is worth mentioning that the health utility value score is higher than the VAS score. The health utility value is derived from the utility score system, reflecting societal preferences. The VAS score, on the other hand, is the subjective score of the interviewees themselves, which is more reflective of their subjective feelings. Therefore, this study suggests that respondent adults tend to hold a slightly pessimistic attitude toward their HRQoL. Both health utility value and VAS scores exhibited a gradual decline with age. Existing surveys also support the observation that the QOL for older individuals in China is not optimistic (47, 48), aligning with the outcomes of this paper. While some studies indicate that urban adults generally have a higher HRQoL than their rural counterparts (49, 50), the difference in HRQoL between urban and rural adults in this study is small and not statistically significant. This could be attributed to the diminishing gap in living standards between urban and rural areas in recent years in China. Nevertheless, it might also be influenced by sample selection.

The exploration of health dimensions revealed that 39.7% of the adults encountered difficulties in at least one dimension, with the prevalence of difficulties in specific dimensions as follows: “mobility” (10.9%), “self-care” (5.2%), “usual routines” (9.5%), “pain/discomfort” (26.2%), and “anxiety/depression” (27.0%). Notably, the dimensions of “pain/discomfort” and “anxiety/depression” exhibited the highest proportion of challenges, corroborating findings from prior research (51–55). Existing studies have indicated that younger individuals tend to experience higher levels of anxiety, while older individuals are more prone to frequent occurrences of pain or discomfort (11). Despite the presence of a ceiling effect, signifying a persistent high proportion, it is evident that pain and psychological anxiety constitute significant factors influencing the QOL among Chinese adults. In the pursuit of enhancing population health, it is pertinent to direct attention toward addressing issues related to pain and psychological anxiety. Thus, a targeted approach toward improving the health status of these two dimensions, namely “pain/discomfort” and “anxiety/depression,” would be more fitting.

Based on the aforementioned findings, factors such as age, marital status, educational level, diet, sleep quality, psychological well-being, state of mind, and the number of chronic diseases were found to be correlated with HRQoL scores. Age emerged as one of the most prevalent and significant factors associated with HRQoL, consistent with previous research. Marital status was found to be partly associated with the physical and mental health dimensions in HRQoL. Dong et al. (22) found that the QOL of married or cohabiting adults was better than the other groups. However, unmarried individuals may have fewer family burdens, leading to better physical health compared to married individuals. Furthermore, our findings indicate that single and unmarried adults reported a higher QOL compared to their married counterparts. This phenomenon may be attributed to the evolving societal trend of delayed marriage among young individuals. The postponement of marriage allows them to attain financial independence, thereby enhancing their overall QOL.

Educational level might directly or indirectly associate with HRQoL in previous surveys (56). The prevailing belief suggests that individuals with higher education levels experience smoother professional trajectories and possess enhanced psychological adjustment abilities. However, this study deviates from conventional wisdom by identifying lower HRQoL among master’s degree holders compared to those with junior/senior high school or college education, similar to the findings of Dong et al. (22). Yao et al. (51) also confirm that the HRQoL of young academics (with higher education levels) in China is lower than the general population at the same age (9). This discrepancy may be attributed to the nature of work undertaken by individuals with master’s degrees and above, often involving more intricate and demanding tasks, leading to heightened psychological and physiological stress. The findings of this study highlight the importance of work-life balance in promoting HRQoL among adults with higher levels of education in China. Building a strong social network may mitigate some of the health risks resulting from high work pressures, but a fundamental solution requires a systems approach. This includes, but is not limited to, fostering a strong caring culture and implementing supportive measures (15).

Mood remains a risk factor affecting the adults’ QOL, and the more normal and stable the mood, the higher the health utility value of the adults. The mean health utility value of pessimistic, all-embracing adults is only 0.6578, which is the lowest of all the entries in this study, and it significantly impacts the HRQoL of the adults. Chronic disease is a risk factor affecting adults’ HRQoL (62), and it has been found that people who are in a chronic state for a long time have a worse HRQoL (23). Chronic diseases are diseases with a long course and complex causes, which impact patients’ QOL, especially for patients with a combination of multiple chronic diseases and older age (57–59). This study showed that having two or more chronic diseases resulted in a decline compared to adults without the disease, consistent with existing results. Beyond the pronounced effects on physical health, chronic diseases also pose a significant threat to patients’ psychological well-being. It is crucial to address both the physical and mental aspects of patients with chronic diseases, offering timely psychological counseling. Hospitals, communities, and other healthcare institutions can organize regular psychological lectures to assist patients in maintaining an optimistic attitude toward their treatment, thereby enhancing their overall QOL.

In relation to sleep, adults experiencing difficulties falling asleep or frequent awakenings reported a significantly lower QOL compared to those with normal sleep patterns, aligning with common perceptions. Additionally, studies have indicated that daytime dysfunction and sleep disorders serve as risk factors for HRQoL across various age groups (60). Notably, middle-aged individuals are more inclined to resort to sleep aids to address sleep-related issues, a practice that may lead to long-term drug dependence and adverse health effects. Research by Japanese scholars has further demonstrated that subjective sleep quality is closely associated with both physical and mental QOL, while sleep duration is specifically linked to mental well-being (60). Consequently, sleep quality emerges as a pivotal indicator of an individual’s overall QOL. Individuals are encouraged to evaluate their sleep conditions, cultivate healthy sleep habits, and seek medical intervention when necessary. Turning attention to dietary habits, adults with reduced appetite and food intake exhibited lower QOL compared to those adhering to a normal diet. Adopting a diet low in oil, salt, and sugar, while ensuring balanced nutrition, has been shown to significantly improve the HRQoL of Chinese adults (61). This underscores the importance of dietary choices in influencing overall well-being, emphasizing the need for individuals to be mindful of their dietary patterns, ensuring a well-balanced and nutritious intake for optimal QOL.

While this study surveyed and analyzed adults from different regions of China, certain limitations emerged due to constraints in research time and effort. Subsequent research endeavors could extend beyond these constraints, facilitating a comparison of data across multiple provinces and years. This broader approach would unveil macroscopic trends, providing insights into the changes in HRQoL among the overall population of China and the underlying influencing factors. Such comprehensive research initiatives will play a pivotal role in advancing the field of HRQoL. They have the potential to yield milestone effects, not only enhancing the HRQoL of Chinese adults but also contributing significantly to the realization of the overarching goal of a healthy China.

## 5 Conclusion

The overall HRQoL among Chinese adults is generally commendable. However, according to our findings, public health strategies to improve HRQoL should be developed to promote relatively healthy environments and lifestyles for the older adults, especially those with chronic diseases. The nation should fortify the screening processes for chronic diseases, implementing comprehensive measures to identify and manage these health conditions effectively. Furthermore, enhancing the overall management and service standards for chronic diseases is paramount. This proactive approach will not only mitigate the adverse effects on HRQoL but also align with the evolving healthcare needs of our aging population.

Although the HRQoL of Chinese adults is favorable, a vigilant focus on health status across all dimensions is imperative, with particular attention warranted in areas such as pain and psychological anxiety, chronic diseases, and negative emotions. These aspects serve as prominent risk factors contributing to the decline in HRQoL among adults. Additionally, we found that normal appetite and food intake, as well as normal sleep conditions, were identified as protective factors for HRQoL. It is suggested to enhance the health awareness of the adults, guide their health behavior and lifestyle, reduce the occurrence of chronic diseases, and improve the QOL from the source.

These outcomes suggest that factors such as the stress associated with marriage and the demands of high-skilled occupations might exert an influence on the overall health of the population. This highlights the need for careful consideration and attention to the well-being of individuals facing these stressors. Consequently, this demographic should be encouraged to adopt stress-relief strategies, prioritize sufficient sleep, and cultivate an optimistic outlook on life. These proactive measures are crucial for enhancing their QOL and mitigating the potential health impacts associated with marital stress and high-skilled employment.

This study provided HRQoL scores and their determinants among Chinese adults, which are valuable for policymakers. Indeed, having an accurate perspective of the societal health status aids the decision-making of planners and policymakers. The main limitation of the present study is the small sample size from Central and Western regions. To overcome these limitations, future studies should include a larger number of residents of Central and Western regions to compare the HRQOL and its determinants among different areas.

## Data Availability

The raw data supporting the conclusions of this article will be made available by the authors, without undue reservation.
